# Semantic-cultural validation and internal consistency analysis of the Purpose in Life Scale for brazilian older adults

**DOI:** 10.1590/1980-57642018dn12-030004

**Published:** 2018

**Authors:** Cristina Cristovão Ribeiro, Anita Liberalesso Neri, Mônica Sanches Yassuda

**Affiliations:** 1 Gerontology, Faculty of Medical Sciences, State University of Campinas, UNICAMP, Campinas, SP, Brazil.; 2 Physiotherapy, CESUFOZ, Center for Higher Education of Foz do Iguaçu, Foz do Iguaçu, PR, Brazil.; 3 Gerontology, School of Arts, Sciences and Humanities, University of São Paulo, SP, Brazil.

**Keywords:** older adults, longevity, well-being, goal-directedness, adultos mais velhos, longevidade, bem-estar, direcionamento de metas

## Abstract

Purpose in life is one of the six dimensions of psychological well-being. It refers to the sense that life has meaning and direction, and that goals have been achieved or are attainable.

**Objective:**

To perform the semantic-cultural validation and internal consistency analysis of the 10-item Purpose in Life scale of Ryff and Keyes.

**Methods:**

Data were drawn from an eight-year follow-up of older adults aged ≥80 in the FIBRA Study, conducted in Campinas, Brazil.

**Results:**

The mean age of participants (N=187) was 83.81 (±3.60), mean number of years of education was 4.38 (±3.76), and mean income was 3.49 minimum wages (±2.61), comprising 125 (66.8%) females (mean purpose = 3.51 ±0.68) and 62 (33.2%) males (mean purpose = 3.58±0.60). There was no significant difference in the purpose between men and women or between educational levels. For age and income, purpose was higher in the 80- to 84-year-old group (younger age) and with income of 3-5 minimum wages (higher income). Cronbach’s alpha for the scale was 0.628, indicating moderate internal consistency.

**Conclusion:**

The Purpose in Life scale was translated, adapted for use in Brazil and applied in a sample of old-old adults. Purpose seemed to be influenced by age and income.

## Resumo

Propósito de vida é uma das seis dimensões do bem-estar psicológico. Refere-se ao senso de que a vida tem sentido e direção e que as metas estão sendo alcançadas.

**Objetivo:**

Realizar a validação semântico-cultural e análise da consistência interna da escala de Propósito de Vida de Ryff e Keyes com 10 itens.

**Métodos:**

Os dados são do seguimento após oito anos do Estudo Fibra, com idosos com 80 anos e mais, realizado em Campinas-Brasil.

**Resultados:**

A média da idade dos participantes (N=187) foi de 83,81 (±3,60), média de anos de escolaridade de 4,38 (±3,76) e renda média de 3,49 salários mínimos (±2,61), sendo 125 (66,8%) do sexo feminino (média de propósito = 3,51 e ±0,68) e 62 (33,2%) do masculino (média de propósito = 3,58 e ±0,60). Não houve diferença significativa no escore de propósito entre os homens e mulheres e entre faixas de escolaridade. Para idade e renda, PV foi mais elevado no grupo com 80 a 84 anos (com menor idade) e com maior renda entre 3 a 5 salários mínimos (maior renda). O Alfa de Cronbach foi 0,628, indicando consistência interna moderada.

**Conclusão:**

A escala de Propósito de Vida foi traduzida, adaptada para uso no Brasil e aplicada em uma amostra de adultos velhos. Propósito parece ser influenciado por idade e renda.

Physical, psychological and social changes that occur in old age call for the need to study the variables that favor the health of older people and successful aging. This is especially true for subjective and psychological well-being.^1^

Subjective well-being (SWB) can be defined as the result of a person’s evaluation of his/her own life.^2^ In turn, psychological well-being (PWB) is a construct that encompasses concepts of human development psychology, humanistic-existential psychology, and positive psychology knowledge about positive or optimal psychological functioning.^3^

According to Ryff and Keyes,^4^^,^ 5 there are six dimensions of PWB: autonomy (independence and self-determination capacity), environmental domain (ability to manage the world around), personal growth (being open to new experiences), positive relationships with others, self-acceptance (positive attitude toward oneself), and purpose in life.

Purpose in life is described by Ryff^6^ as one of the main domains of PWB because it refers to the feeling that life has direction and that goals are attainable, either in the short-, medium- or long-term. Moreover, purpose is related to a more positive view of life, the perception of personal growth, happiness, satisfaction, self-esteem, motivation to live and to perform daily activities.^3^

To the best of our knowledge, this is the first study carried out about purpose in life involving Brazilian older adults. Given that purpose in life is amenable to psychological intervention,^6^ it seems relevant to validate a scale that allows its assessment in Brazil. The achievement of this objective may favor research and clinical and educational interventions in the context of aging in Brazil. Therefore, the aim of the present study was to perform the semantic-cultural validation and the internal consistency analysis of the 10-item Purpose in Life scale developed by Ryff and Keyes in a Brazilian sample of old-old adults.

## METHODS

The study was carried out in two phases: a semantic-cultural validation and an assessment of the internal consistency of the scale.

### Phase 1: Semantic-cultural validation of the translation of the Purpose in Life scale for Brazil

The procedures described by Reichenheim and Moraes^7^ for the translation process, back-translation and semantic-cultural adaptation were adopted. In the first step, the original Purpose in Life scale was translated into Brazilian Portuguese by two bilingual individuals who worked independently. In the second step, a synthesis of the first Portuguese version was prepared by a specialist in Geropsychology, who produced the first version of the scale. The third step was involved back-translation by two other bilingual individuals, who were native English speakers. They worked independently, with no information on the underlying theory of the scale and no access to the version published in English. After analysis of the back-translations and comparisons with the original scale, a final version was produced, for which a 100% consensus was attained between translators and back-translators.

### Phase 2: Internal consistency analysis of the Purpose scale and preliminary analyses regarding the effects of sex, education, age and income

The version translated and adapted to Portuguese of the Purpose in Life scale was included in the follow-up protocol of the FIBRA Study (2008-2009)^8^ which was used to assess 187 older adults aged 80 years or older in both 2016 and 2017. All participants signed an informed consent form approved by the CEP/Unicamp on December 10, 2014 (CAAE 39547014.0.1001.5404).

The Mann-Whitney test was used to compare the scores of the Purpose in Life scale between sexes, and the Kruskal-Wallis test was used to compare scores among education, age and income bands given the non-normal distribution of variables. The Dunn post hoc test was used after the Kruskal Wallis test to verify which groups differed.

The level of significance was set at 0.05. Statistical analyses were performed using SAS (Statistical Analysis System) for Windows (version 9.2). The reliability of the scale was verified by IC analysis, indicated by the Cronbach’s alpha coefficient.^9^^,^ 10

## RESULTS

### Phase 1: Steps for producing the Brazilian version of the Purpose scale

Subtle cultural differences were observed for some of the scale items, except for item 4, but the semantic equivalence with the original version was not affected. The final version was obtained through consensus among the translators (see Table 1).

**Table 1:** Versions of the Purpose in Life scale throughout the steps of semantic-cultural validation to Brazilian Portuguese. Campinas, SP, 2018.

Items	Original	Translation	Back-translation	Final version
1	I feel good when I think of what I have done in the past and what I hope to do in the future.	**T1:** Eu me sinto bem quando eu penso nas coisas que fiz no passado e o que espero fazer no futuro. **T2:** Eu me sinto bem quando eu penso nas coisas que fiz no passado e o que espero fazer no futuro.	**R1:** I feel fine when I think about the things I have done in the past and what I wish to do in the future. **R2:** I feel good when I think of the things I’ve done in the past and what I hope to do in the future.	Eu me sinto bem quando penso nas coisas que fiz no passado e nas que espero fazer no futuro.
2	I live life 1 day at a time and do not really think about the future.	**T1:** Eu vivo um dia de cada vez e não penso sobre o futuro. **T2:** Eu vivo a vida um dia de cada vez e realmente não penso sobre o futuro.	**R1:** I live one day at a time and I don’t think about the future. **R2:** I live life one day at a time and don’t really think about the future.	Eu vivo a vida um dia de cada vez e realmente não penso sobre o futuro.
3	I tend to focus on the present because the future nearly always brings me problems.	**T1:** Eu foco no presente pois o futuro quase sempre me traz problemas. **T2:** Eu prefiro focar no presente, porque o futuro quase sempre me traz problemas	**R1:** I focus on the present because the future often brings me problems. **R2:** I’d rather focus on the present because the future almost always gets me into trouble.	Eu prefiro focar no presente, porque o futuro quase sempre me traz problemas.
4	I have a sense of direction and purpose in life.	**T1:** Eu tenho senso de direção e propósito na vida. **T2:** Eu tenho um senso de direção e de propósito na vida.	**R1:** I have a sense of direction and purpose in life. **R2:** I have a sense of direction and purpose in life.	Eu tenho um senso de direção e propósito de vida.
5	My daily activities often seem trivial and unimportant to me.	**T1:** Minhas atividades diárias com frequência parecem banais e irrelevantes para mim. **T2:** Minhas atividades diárias com frequência me parecem banais e sem importância.	**R1:** My daily tasks often seem trivial and irrelevant to me. **R2:** My everyday activities often seem trivial and unimportant to me.	Minhas atividades diárias com frequência me parecem banais e sem importância.
6	I used to set goals for myself, but that now seems like a waste of time.	**T1:** Eu costumava ter metas para mim, mas agora parece perda de tempo. **T2:** Eu costumava estabelecer metas para mim, mas agora isso me parece uma perda de tempo.	**R1:** I used to have goals for myself, but now it seems like a waste of time. **R2:** I used to set goals for myself, but now that seems like a waste of time.	Eu costumava estabelecer metas para mim, mas agora isso me parece uma perda de tempo.
7	I enjoy making plans for the future and working them to a reality.	**T1:** Eu gosto de fazer planos para o futuro e trabalhar para torná-los realidade. **T2:** Eu gosto de fazer planos para o futuro e trabalhar para torná-los realidade.	**R1:** I like to make plans for the future and to work so they become true. **R2:** I like to make plans for the future and work to make them come true.	Eu gosto de fazer planos para o futuro e trabalhar para torná-los realidade.
8	I am an active person in carrying out the plans I set for myself.	**T1:** Eu sou uma pessoa ativa na execução dos planos que eu estabeleci para mim mesmo. **T2:** Eu sou uma pessoa ativa na execução dos planos que eu estabeleci para mim mesmo.	**R1:** I am an active person in the execution of the plans I have stablished for myself. **R2:** I’m an active person in carrying out the plans I’ve made for myself.	Eu sou uma pessoa ativa na execução dos planos que eu estabeleci para mim mesmo.
9	Some people wander aimlessly through life, but I am not one of them.	**T1:** Algumas pessoas vagam sem rumo pela vida, mas eu não sou uma delas. **T2:** Algumas pessoas vagam sem objetivo pela vida, mas eu não sou uma delas.	**R1:** Some people wander aimlessly through life, but I am not one of them. **R2:** Some people wander through life without a purpose, but I’m not one of them.	Eu não sou como algumas pessoas que parecem vagar sem objetivo de vida.
10	I sometimes feel as if I have done all there is to do in life.	**T1:** Às vezes sinto que eu já fiz tudo na vida. **T2:** Às vezes eu me sinto como se já tivesse feito tudo na vida.	**R1:** Sometimes I feel like I have done everything in life. **R2:** Sometimes I feel as if I’ve done everything there is to do in life.	Às vezes eu me sinto como se játivesse feito tudo na vida.

Note: It is a Likert-type scale, anchored by the expressions: (1) Não concordo de jeito nenhum; (2) Concordo pouco; (3) Concordância moderada; (4) Concordo muito; (5) Concordo muitíssimo.

The Purpose in Life scale is a self-report measure with 10 items (Table 1). It is a Likert-type scale, anchored by the expressions: [1] I strongly disagree (Não concordo de jeito nenhum); [2] I agree a little (Concordo pouco); [3] Moderate agreement (Concordância moderada); [4] I Agree a lot, (Concordo muito); [5] I strongly agree (Concordo muitíssimo). To calculate the final score, it is necessary to reverse the score negatively for the items 2, 3, 5, 6 and 10.^11^ The final score is a result of the average of the answers to the 10 questions (sum/10), which can range from 1 to 5.

### Phase 2: Internal consistency analysis of the purpose scale and preliminary analyses regarding the effects of sex, education, age and income

Of the total participants (n=187), 125 (66.8%) were women. Age ranged from 80 to 98 years, with a mean of 83.81 (±3.60); 124 older adults were aged between 80 and 84 years, 45 between 85 and 89 years, while 18 were aged 90 years and over. The mean number of years of education was 4.38 (±3.76), ranging from 0 to 9 years, and 13.89% of the sample had no formal schooling, 60.56% had 1 to 4 years of schooling, 15.56% had 5 to 8, and 10% had 9 years or more. The average income was 3.49 minimum wages (MW) (±2.61), 9.76% earned less than 1 MW, 47.56% earned from 1 to 3 MW, 20.73% earned 3 to 5 MW, 18.90% earned 5 to 10 MW and 3% had an income greater than 10 MW. Among women, the mean Purpose in Life score was 3.51 points (±0.68) whereas among men, the mean was 3.58 points (±0.60). There were no statistically significant differences in scores between sexes or educational levels. Regarding age and income, purpose was higher in the 80- to 84-year-old group and among those with an income of between 3 and 5 MW (Table 2).

**Table 2:** Purpose in Life scores according to sociodemographic variables. Campinas, SP, 2018.

Variable		Gross frequency	Percentage frequency	Mean (SD) for Purpose in Life	p-value
Age	80-84	124	66.3	3.62 (0.61)	0.036^a^
	85-89	45	24.0	3.36 (0.78)	
	90+	18	9.7	3.36 (0,47)	
Sex	Male	62	33.2	3.58 (0.60)	0.781
	Female	125	66.8	3.51 (0.68)	
Education	Illiterate	25	12.4	3.46 (0.59)	0.231
	1-4	116	62.0	3.48 (0.69)	
	5-8	28	15.6	3.59 (0.51)	
	9+	18	10	3.84 (0.68)	
Income (R$ 954.00)	0-1	16	9.8	3.26 (0.87)	0.026^b^
	1.1-3.0	85	45.4	3.49 (0.63)	
	3.1-5.0	36	19.2	3.81 (0.43)	
	5+	50	25.6	3.47 (0.60)	

a 80-84 ≠ 90+;

b 0-1 ≠ 3.1-5 MW.

MW: minimum wage. P value refers to the Mann-Whitney and Kruskal Wallis test. The Dunn post hoc test was used after the Kruskal Wallis test to verify which groups differed.

The acceptable values of Cronbach’s alpha coefficient vary among authors, but in general, we can consider α ≤0.30 = very low IC or reliability; 0.30 < α ≤ 0.60 = low; 0.60 < α ≤ 0.75 moderate; 75 < α ≤ 0.90 = high; and α > 0.90 = very high.^12^

The degree of internal consistency of the Purpose in Life scale was 0.628, as indicated by Cronbach’s alpha, thus suggesting moderate internal consistency.

## DISCUSSION

The present study represents an attempt to advance the research on purpose in life by means of the semantic-cultural validation and internal consistency analysis of a scale. The translation of the 10-item Purpose in Life scale developed by Ryff and Keyes^5^ was carried out, producing its Brazilian Portuguese version. Internal consistency analysis suggested that the scale produced has moderate consistency. Purpose was higher among those with lower age (80 to 84 years) and with higher income (3 to 5 MW).

The current results regarding the relationship between purpose and age are consistent with previous findings of Ryff and Keyes,^5^ Verduin et al.^13^ and Kim et al.^14^ in which purpose scores were also higher among younger older adults.^3^ It can be assumed that younger elderly have better health and functional status, which in turn can contribute to the devising of goals and objectives.^13^ On the other hand, results may suggest that in advanced age- 85 years or older - there may be restriction in plans and accomplishment of goals, as this is a more vulnerable group. Moreover, older adults may perceive their life time as shorter, making the idea of achieving goals unfeasible, therefore affecting purpose.^9^

The effect of age on purpose may be associated with a reduction in positive coping strategies (optimism) and the absence of a social support network, favoring social isolation.^6^ Aging may increase the cognitive, social and physical losses such as widowhood, retirement, loss of occupational roles, loss of loved ones, limited mobility, and disability.^15^ These, in turn, may decrease the engagement of older people in activities.

According to Scheier et al.,^16^ engagement in activities correlates with a variety of other psychosocial factors such as optimism, social network size, self-assessment of health, and physical and mental functioning. These psychosocial and physical aspects tend to decline in older people and may be associated with decreased purpose in life.

The Purpose in Life score was higher in the group with higher income, a phenomenon also observed in a recent study by Hill et al.^17^ In the latter study, the authors suggested that purposeful individuals tend to be more focused on their occupational goals and to pursue their long-term goals, thus striving for career success, which would likely increase personal income. It is also possible to speculate that people with higher income may have the necessary conditions to have more ambitious goals, which could be reflected positively in measures of purpose. It is noteworthy that those with the highest income did not have the highest Purpose in Life score. It is possible that the largest differences are observed between those that have very limited resources (0-1 MW) and those with higher income (3.1 to 5 MW). After this income level, financial resources may be less influential on purpose. Alternatively, Purpose in Life scores may have reached a plateau for this sample of old-old individuals.

There was no significant difference in the Purpose in Life score between men and women and no consensus has been reached regarding differences in purpose between men and women. In the study by Boyle et al.,^11^ women had higher purpose when compared to men, different from the findings of Hedberg et al.,^18^ in which women scored lower than men. In the studies by Kim et al.,^14^^,^ 19 and Boyle et al.,^20^ the results were similar to the findings of the present study, as there was no difference in the purpose in life of men and women. This inconsistency may be associated with differences in the sociodemographic characteristics of the samples studied, such as income and age.

With regard to education, the above-mentioned studies were unanimous in reporting that Purpose in Life scores did not vary among individuals with different educational levels. However, according to the findings of Ryff and Singer,^21^ psychological well-being and education are strongly linked, especially in the domains of personal growth and purpose in life. According to the authors, opportunities for personal accomplishment are not equally distributed. It is assumed that higher educated individuals are more likely to put their skills into practice, increasing self-accomplishment, psychological well-being, and reflecting favorably on purpose scores.

Regarding the internal consistency of the scale, previous studies^11^^,^ 14 ^,^ 22 ^,^ 23 using the Purpose in Life scale also found moderate consistency values, similar to those found in the current study. This finding may be associated with the fact that this version of the scale contains a small number of questions (10 items).

As a limitation of the present study, we highlight that the sample comprised individuals older than 80 years with good functional status and cognition, which may have limited the variability of responses on the Purpose in Life scale. This feature may restrict possible generalizations, for example, for younger older adults.

In summary, the semantic-cultural validation and internal consistency analysis of the Purpose in Life scale were carried out. Future studies involving heterogeneous samples of older adults are necessary. However, the present study has merits as it evaluated purpose in life among individuals aged 80 years and older and provided the semantic-cultural validation and internal consistency analysis for the scale. We hope that this validation may stimulate further research on this theme and promote interventions to increase purpose in life among older adults. Gerontological research should produce knowledge about aging in advanced ages, especially regarding the variables that can contribute to healthy longevity.
